# Outcomes of children with cystic fibrosis screen positive, inconclusive diagnosis/CFTR related metabolic syndrome

**DOI:** 10.3389/fped.2023.1127659

**Published:** 2023-03-09

**Authors:** Mohini A. Gunnett, Elizabeth Baker, Cathy Mims, Staci T. Self, Hector H. Gutierrez, Jennifer S. Guimbellot

**Affiliations:** ^1^ Department of Pediatrics, Division of Pulmonary and Sleep Medicine, University of Alabama at Birmingham (UAB), Birmingham, AL, United States; ^2^ Gregory Fleming James Cystic Fibrosis Research Center, University of Alabama at Birmingham (UAB), Birmingham, AL, United States; ^3^ Department of Sociology, University of Alabama at Birmingham (UAB), Birmingham, AL, United States

**Keywords:** CF, cystic fibrosis, CFTR (cystic fibrosis transmembrane conductance regulator), NBS (Newborn screening), SCT (sweat chloride testing), CFSPID/CRMS, CF screen positive, inconclusive diagnosis (CFSPID)/CFTR-related metabolic syndrome (CRMS)

## Abstract

**Background:**

Some infants undergoing newborn screening (NBS) tests have inconclusive sweat chloride test (SCT) results that lead to the designation of Cystic Fibrosis Screen Positive, Inconclusive Diagnosis/CFTR-related metabolic syndrome (CFSPID/CRMS). Some proportion of them transition to a CF diagnosis, but no predictive markers can stratify which are at risk for this transition. We report single-center outcomes of children with CRMS.

**Methods:**

We retrospectively identified all infants born in Alabama from 2008 through 2020 referred to our CF Center with an elevated immunoreactive trypsinogen level (IRT) associated with a cystic fibrosis transmembrane conductance regulator (CFTR) mutation (IRT+/DNA+) who had at least one SCT result documented. Infants were classified per established guidelines as Carrier, CRMS, or CF based on the IRT+/DNA+ and SCT results. The electronic health record was reviewed for follow-up visits until the children received a definitive diagnosis (to carrier or CF) according to current diagnostic guidelines for CF, or through the end of the 2020 year.

**Results:**

Of the 1,346 infants with IRT+ and at least 1 CFTR mutation identified (IRT+/DNA+), 63 (4.7%) were designated as CRMS. Of these infants, 12 (19.1%) transitioned to Carrier status (CRMS-Carrier), 40 (63.5%) of them remained CRMS status (CRMS-Persistent) and 11 (17.5%) of them transitioned to a diagnosis of CF (CRMS-CF). Of the 11 children in the CRMS-CF group, 4 (36%) had an initial SCT 30–39 mmol/L, 4 (36%) had an initial SCT 40–49 mmol/L and 3 (27%) had an initial SCT 50–59 mmol/L. These children also had higher initial sweat tests and greater yearly increases in sweat chloride values than others with CRMS. We found that in comparison to children in the CRMS-*P* group, a greater proportion of children in the CRMS-CF group cultured bacteria like methicillin-resistant *Staphylococcus aureus, Stenotrophomonas maltophilia, and Pseudomonas aeruginosa,* had smaller weight-for-height percentiles and remained smaller over time despite slightly greater growth.

**Conclusion:**

Infants with an inconclusive diagnosis of CF should continue to receive annual care and management given their potential risk of transition to CF. Further research is needed to assess whether certain phenotypic patterns, clinical symptoms, diagnostic tests or biomarkers could better stratify these children.

## Introduction

Cystic fibrosis (CF) is an autosomal recessive genetic disorder caused by mutations in the CF transmembrane conductance regulator (CFTR) gene (1). Early identification of individuals with CF is critical towards improved growth and development, as well as survival (1, 2). Implementation of newborn screening (NBS) nationally has led to earlier detection of patients with CF and better outcomes for this patient population (3).

NBS programs across the world use a variety of algorithms for screening. Most begin with the measurement of immunoreactive trypsinogen (IRT), which is produced by the pancreas and elevated in the blood of infants with CF (4). In Alabama, newborns with an elevated IRT on NBS will then obtain a screening CFTR gene mutation analysis using a commercially available panel (5, 6). In general, newborns with an elevated IRT and at least one mutation identified on the genetics panel are considered to have a positive NBS and are immediately referred for confirmatory testing with a sweat chloride test (SCT). An infant with a normal sweat chloride (SC) concentration <30 mmol/L and only one mutation is designated a carrier, whereas an infant with a SC concentration ≥ 60 mmol/L and/or two known CF disease-causing mutations confirms a definitive diagnosis of CF (7).

Advent of the NBS has led to the identification of asymptomatic infants found to have a positive NBS with at least one mutation and a SC concentration between 30 and 59 mmol/L, who are considered to have an inconclusive diagnosis and may be designated to have CF Screen Positive, Inconclusive Diagnosis (CFSPID), also known as CFTR-related metabolic syndrome (CRMS) (8–15). Repeated SCT in the diagnostic range or identification of two known CF disease-causing mutations can subsequently confirm the diagnosis of CF (15, 16). Some children do not develop diagnostic range SCT (14–17). Children may also present with one or more mutations in CFTR of variable clinical consequence (VCC), which may result in symptomatic CF but are also found in healthy individuals who do not develop any symptoms of disease (5, 7). Typically, children who carry the designation of CRMS remain free of symptoms for years and may never develop symptoms consistent with a CF diagnosis. However, several longitudinal studies have noted anywhere from 5% to 48% of these children will develop clinical features suggestive of CFTR-related disorder (CFTR-RD) or CF (5, 7, 13, 16–20). Despite this notable risk of transition, there are no clear predictive biomarkers to identify which children with CRMS have a higher risk of latter transitioning to a diagnosis of CF disease. A few global studies have demonstrated a potential association between initial IRT values and genetics that predict transition to a CF diagnosis (21–24). Two retrospective studies noted a higher likelihood of transition to CF if an infant's initial SCT value was between 50 and 59 mmol/L (15) or if SCT progressed toward diagnostic range faster (24). These biochemical markers are not yet part of guidelines for CRMS management and are challenging to use on an individual basis to counsel families. Furthermore, current guidelines remain unclear on the frequency and duration of follow-up for patients with CRMS (16). In this study, we report the outcomes of children with CRMS after implementation of NBS in the only accredited CF center in the state, to further add to the current literature on this topic and explore novel approaches to next steps for guideline development.

## Methodology

### State protocol and confirmatory testing

The state of Alabama uses an IRT/DNA protocol that involves a 2-step process of measuring IRT followed by screening for cystic fibrosis transmembrane regulator (CFTR) mutations on a limited DNA panel for any infants with an IRT value >96th percentile on that designated day (6). The NBS is considered positive if an infant has IRT value >96th percentile AND carries at least 1 mutation identified on the DNA screening panel or if the infant has an IRT value >99.9th percentile for the day. The Alabama Department of Public Health used InPlex® CF Molecular Test (Hologic, Ltd.) until August 2016, then changed to the xTAG® Cystic Fibrosis (CFTR) 39 v2 K (Luminex Corporation, Austin, Texas, United States.) A small number of newborns are referred for other reasons such as a very high IRT/failed NBS, family history of CF, or other reasons for concern. Any NBS-positive infants then undergo confirmatory SCT at our CF-accredited Center here at the University of Alabama at Birmingham (UAB) within Children's of Alabama (CoA) Hospital. Additional genetic analysis (CFTR sequencing with deletion/duplication analysis) was performed at provider discretion (persistently intermediate SCT or heightened clinical suspicion). The combination of the information provided by the NBS and confirmatory SCT provides the infant with their initial classification (per the most recently updated guidelines (13)) of one of the following: non-carrier (SCT <30 mmol/L and no CFTR mutations), carrier status (SCT <30 mmol/L and one CFTR mutation), CF (SCT ≥60 mmol/L and/or two disease-causing mutations), or CRMS (indeterminate SCT 30–59 mmol/L and less than two disease-causing mutations OR SCT <30 mmol/L and two CFTR mutations with at least one of unclear phenotypic consequences).

### Study population

This study was approved by the UAB Institutional Review Board (#300005123). We retrospectively reviewed the charts of all newborns born in Alabama between the years 2008 to 2020 referred to our center for evaluation of cystic fibrosis. Initial newborn screening results, including IRT value and mutation screening results, were collected for each of these infants. Data collected from available charts for any follow-up visits through January 2021 included self-reported race/ethnicity, sex, first quantifiable SCT, CFTR genetic results, microbiology results from sputum swabs, stool elastase, lung function, weight, and height measures. Infants initially designated CRMS were sub-classified by final diagnosis when data collection ended. A child transitioned to carrier status (CRMS-Carrier) with normalized SCT and if only one CFTR mutation identified. If a child met criteria for CF diagnosis [4] with repeat SCT concentration resulted >60 mmol/L, expanded genetics revealed a second CF-causing mutation, development and persistence of clinical symptoms, or with re-classification of their VCC mutation to a CF-causing mutation (determined by CFTR2 project database (25)), then they transitioned to a CF diagnosis (CRMS-CF). All other newborns who remained inconclusive diagnosis remained in the CRMS category as CRMS-Persistent (CRMS-*P*). Children designated CRMS are followed by pediatric pulmonologists with CF expertise in a separate clinic space than that of children followed in our CF clinics.

### Statistical analysis

Descriptive analyses (means, standard deviations, medians, interquartile ranges, frequency distributions (%)) were used to describe patient demographics, clinical characteristics, and outcomes. Results are presented as means +/− standard deviation (SD) or standard error of the mean (SEM) where noted. For all analyses, a *p*-value of <0.05 was considered statistically significant. We compared initial diagnoses (CF, CRMS, Carrier) and CRMS subgroups (CRMS-CF, CRMS-Carrier, and CRMS-*P*) using analysis of variance. Tukey's adjustment for multiple comparisons was completed for each comparison. Anthropomorphic trajectories and SCT trajectories are modeled using multilevel linear mixed models with a random intercept. Change over time is modeled as age. Statistical analysis was performed using STATA software (StataCorp,College Station, Texas) and GraphPad Prism (San Diego, California).

## Results

### Demographics of NBS population

**Figure 1:**
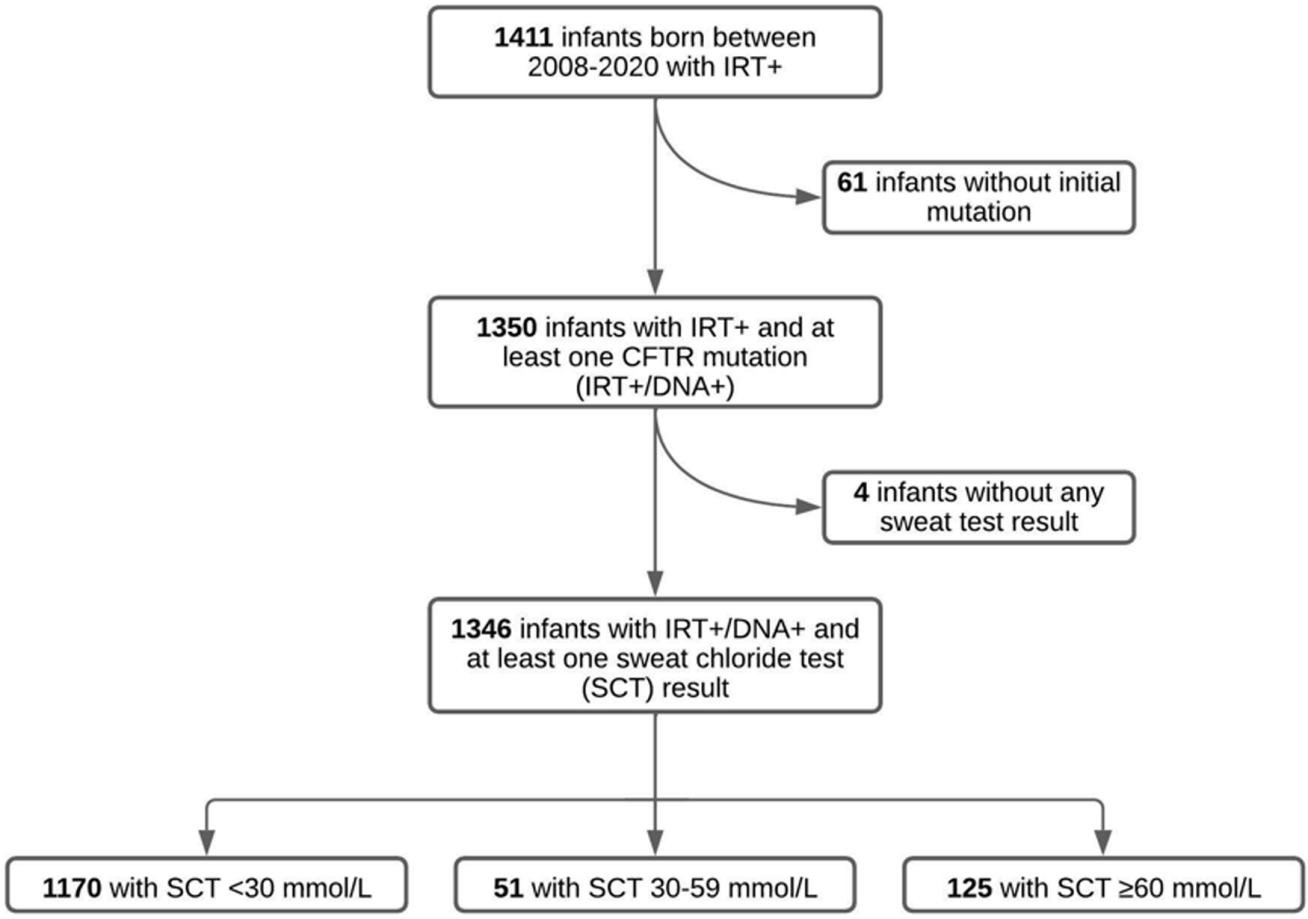
Results of SCT in infants referred for elevated IRT and 1 mutation (IRT+/DNA+) in Alabama between 2008 and 2020. Of the initial 1,411 infants referred for elevated IRT, 65 of them were excluded due to not having an initial CFTR mutation or a documented SCT. Our study population therefore included 1,346 infants with IRT+/DNA+ screening and at least one successful SCT concentration.

From 2008 to 2020, there were 1,411 infants identified through NBS with elevated IRT (IRT+) and referred to our CF Center. Sixty-one infants with no CFTR mutation identified on screening genetics and a normal SCT did not undergo any further evaluation, and were therefore excluded from the study. Four infants had insufficient quantities on all SCT attempts in the record and were excluded from the study. The remaining 1,346 infants with IRT+ and ≥1 CFTR mutation identified (IRT+/DNA+) had at least one SCT result documented. Of these 1,346 infants, 1,170 infants (86.9%) had an initial SC concentration <30 mmol/L, 51 infants (3.8%) had an intermediate SC concentration between 30 and 59 mmol/L and 125 infants (9.3%) had an initial SC concentration ≥ 60 mmol/L (Figure 1).

Characteristics for all infants who presented with IRT+/DNA+ and confirmatory SCT are shown in Table 1. All 125 infants with SC concentrations >60 mmol/L were classified as CF on initial evaluation. Four infants with initial intermediate SC concentrations had 2 CF-causing mutations result on their NBS DNA mutation panel and were therefore classified as CF on their initial diagnosis. Forty-seven infants with initial intermediate SC concentrations met the criteria for CRMS classification. Of the 1,170 infants with initial SC concentrations <30 mmol/L, 16 resulted with 1 CF-causing mutation and 1 variant of VCC mutation on their NBS DNA panel and were therefore classified as CRMS on initial evaluation. The remaining 1,154 infants with initial SC concentrations <30 mmol/L had only one mutation identified and were classified as Carriers. Therefore, of the total 1,346 infants included in our study analysis, 129 infants (9.6%) were classified as CF on their initial assessment, 63 (4.7%) as CRMS, and 1,154 (86%) as Carriers. This indicates a CF:CRMS ratio of 2.1:1.0. As consistent with known data on IRT in newborns with CF, we found newborns diagnosed with CF after completing the NBS process had a higher IRT and initial SCT value when compared to children initially designated as CRMS (*p *<* *0.001 for IRT; *p *<* *0.0001 for SCT) or Carrier (*p *<* *0.001 for IRT; *p *<* *0.001 for SCT, data not shown). There was also a statistically significant difference seen in children with initial designations of CRMS compared to Carrier for IRT values (*p *=* *0.0281) and first SCT value (*p *<* *0.0001).

**Table 1:** Characteristics for all infants with IRT+/DNA+ and at least one successful SCT (*n *= 1,346).

	All population	Carrier	CRMS	CF
*n* (% of total)	1,346	1,154 (86%)	63 (5%)	129 (10%)
Sex
Male: *n* (%)	661 (49%)	561 (49%)	35 (56%)	65 (50%)
Female: *n* (%)	685 (51%)	593 (51%)	28 (44%)	64 (50%)
Race
Caucasian: *n* (%)	964 (72%)	808 (70%)	47 (75%)	109 (84%)
AA: *n* (%)	296 (22%)	270 (23%)	10 (16%)	16 (12%)
Hispanic: *n* (%)	47 (3.5%)	41 (1.8%)	2 (3.2%)	4 (3.1%)
Biracial: *n* (%)	31 (2.3%)	28 (2.4%)	3 (4.8%)	0
Other: *n* (%)	7 (0.5%)	6 (0.5%)	1 (1.6%)	0
Unknown: *n* (%)	1 (0.07%)	1 (0.08%)	0	0
IRT Value (ng/mL)	75.5 (23–310.5)	66.7 (23–301.4)	78.0 (42.2–162)	158.5 (45.8–310.5)
	(*n *=* *1188)	(*n *=* *1026)	(*n *=* *55)	(*n *=* *107)
Initial SCT (mmol/L)	21.9 (4–122)	13.0 (4–29)	33.7 (12–59)	95.0 (36–122)
	(*n *=* *1346)	(*n *=* *1154)	(*n *=* *63)	(*n *=* *129)
Initial SCT value (mmol/L)
<30: *n* (%)	1,170 (87%)	1,154 (100%)	16 (25%)	0
30–59: *n* (%)	51 (4%)	0	47 (75%)	4 (3.1%)
>60: *n* (%)	125 (11%)	0	0	125 (97%)

### Outcomes of infants initially designated with CRMS

Characteristics of the 63 infants who were classified as CRMS on their initial encounter are shown in Table 2. Of these 63 infants, 16 (25%) had an initial SC value <30 mmol/L and 47 (78%) had an initial intermediate SC value between 30 and 59 mmol/L. Average IRT values for infants classified as CRMS was 75.2 ng/ml. Within the timeframe of the study, 12 (19%) of the total 63 CRMS infants transitioned to Carrier status (CRMS-Carrier), 40 (64%) of them remained CRMS status given persistent inconclusive diagnosis (CRMS-*P*), and 11 (18%) of them transitioned to a diagnosis of CF (CRMS-CF). All 12 CRMS-Carrier infants had an initial SC value in the intermediate range. All 12 of these infants had a repeat SCT that normalized to <30 mmol/L. Average IRT values for infants classified as CRMS-Carrier was 67.4 ng/ml. Of the 40 infants who remained CRMS status by the end of the observation period (CRMS-*P*), 16 (40%) had an initial SC value <30 mmol/L and 24 (60%) had an initial SC value in the intermediate range. Eleven (28%) of these patients were either lost to follow-up or had their care transferred to another CF Center after moving to another state. The remainder 29 (72.5%) patients are followed at our CF center for continued evaluation as per the CRMS guidelines.

**Table 2:** CRMS-*P*, CRMS-Carrier, CRMS-CF diagnosis.

	All CRMS Population	CRMS-*P*	CRMS-Carrier	CRMS-CF
*n* (% of total)	63	40 (63.5%)	12 (19.1%)	11 (17.5%)
Sex
Male: *n* (%)	35 (56%)	25 (62%)	6 (50%)	4 (36%)
Female: *n* (%)	28 (44%)	15 (38%)	6 (50%)	7 (64%)
Race
Caucasian: *n* (%)	47 (75%)	31 (78%)	10 (83%)	6 (54%)
AA: *n* (%)	10 (16%)	5 (12%)	1 (8.3%)	4 (36%)
Hispanic: *n* (%)	2 (3.2%)	1 (3.3%)	1 (8.3%)	0
Biracial: *n* (%)	3 (4.8%)	2 (5%)	0	1 (9%)
Other: *n* (%)	1 (1.6%)	1 (3.3%)	0	0
IRT Value (ng/mL)	78.0 (42.2–162)	75.2 (42.2–157.9)	67.4 (44.5–108.5)	97.8 (43.5–162)
	(*n *=* *55)	(*n *=* *36)	(*n *=* *9)	(*n *=* *10)
Initial SCT (mmol/L)	33.7 (12–59)	30.0 (12–56)	35.0 (30–57)	45.0 (33–59)
	(*n *=* *63)	(*n *=* *40)	(*n *=* *12)	(*n *=* *11)
Median Age at 1st SCT [days, IQR]	30 [22–41]	31 [21–45]	30 [20.3–44.8]	30 [27.5–36]
Mutation on NBS
*F508DEL*	46	31	6	9
*G542X*	1	1	0	0
*G551D*	1	0	1	0
*S549N*	1	1	0	0
*R117H*	1	1	0	0
*R117H 5 T/7T*	1	1	0	0
*R117H 7 T/7T*	2	0	2	0
*R117H 7 T/9T*	2	2	0	0
*5 T;TG13*	1	0	1	0
*R553X*	1	0	0	1
*621 + 1G->T*	1	1	0	0
*3120 + 1G > A*	3	1	1	1
*1717-1G > A*	1	0	1	0
*R1162X*	1	1	0	0
Initial SCT (mmol/L)
<30: *n* (%)	16 (25%)	16 (40%)	0	0
30–59: *n* (%)	47 (75%)	24 (60%)	12 (100%)	11 (100%)
*30–39: n (%)*	34 (54%)	20 (50%)	10 (83%)	4 (36%)
*40–49: n (%)*	8 (13%)	3 (7.5%)	1 (8.3%)	4 (36%)
*50–59: n (%)*	5 (7.9%)	1 (2.5%)	1 (8.3%)	3 (27%)

### Detailed characteristics of children transitioned from CRMS to cf

Diagnostic data for the 11 total infants initially designated with CRMS who had a final diagnosis of CF (CRMS-CF) are shown in Table 3. All 11 CRMS-CF infants had an initial SC value in the intermediate range. Four (36%) of the 11 CRMS-CF infants had an initial SC concentration between 30 and 39 mmol/L, 4 (36%) had an initial SC concentration between 40 and 49 mmol/L and 3 (27%) had an initial SC concentration between 50 and 59 mmol/L. Five (45%) of them were transitioned to a CF diagnosis after repeat SCT resulted >60 mmol/L, 3 (27%) transitioned after development and persistence of clinical symptoms, 2 (18%) transitioned after expanded genetics revealed a second CF-causing mutation and 1 (9%) was re-classified as CF after one of their mutations was re-classified as CF-causing on the CFTR2 project database. Of the 5 children that transitioned to a CF diagnosis after having repeat SC concentrations result positive, 3 (60%) had initial SC concentrations 50–59 mmol/L, 1 (20%) had initial SC concentrations 40–49 mmol/L, and 1 (20%) had initial SC concentrations 30–39 mmol/L. Of the 3 children that transitioned to CF diagnosis after development of clinical symptoms, 2 (67%) had initial SC concentrations 30–39 mmol/L and 1 (33%) had initial SC concentrations 40–49 mmol/L. All 3 of these children were transitioned to a CF diagnosis due to the persistence of clinical symptoms including cough, spirometry changes, and pulmonary exacerbations that were accompanied by persistent methicillin-resistant staphylococcus aureus (MRSA), pseudomonas aeruginosa or non-tuberculous mycobacteria (NTM) culture growth. These 3 children have also demonstrated upward trending SC values on repeat testing. Of the 2 children that transitioned to a diagnosis of CF after expanded genetics workup revealed a second CF-causing mutation, both had an initial SC concentrations 30–39 mmol/L. The 1 infant who transitioned to CF after their mutation (F191V) was classified as CF-causing had an initial SC concentration 40–49 mmol/L. Average IRT for the 11 children with CRMS-CF was 97.8 ng/ml. The average age for these children when they were transitioned to a diagnosis of CF was 3.9 years of age (range 0.18 years of age to 8.4 years of age). Four of these children were transitioned to a diagnosis of CF after the age of 6 years old, with 1 converting after repeat SCT >60 mmol/L, 2 converting after the development and persistence of clinical symptoms, and 1 being re-classified after genetic mutation F191V classified as CF-causing. Patients 3 and 7 are siblings with the same genetic mutations, one of whom transitioned at 8 years old after their fifth SCT was positive at 61 mmol/L and repeat stool elastase found to be at 118, while the other sibling transitioned at 2 years old after developing clinical symptoms of cough, spirometry changes, upward trending SCT values, and oropharyngeal culture growth of methicillin-sensitive *Staphylococcus aureus* (MSSA) and *Pseudomonas aeruginosa* requiring eradication. Three patients had *Pseudomonas aeruginosa* growth on oropharyngeal cultures and 4 patients had MRSA growth on oropharyngeal cultures. None of the children with initial diagnosis of CRMS had evidence of hospitalizations for severe respiratory concerns prior to their transition to a CF diagnosis.

**Table 3:** Diagnostic data for infants transitioned to diagnosis of CF.

Pt. #	IRT (ng/ml)	Initial SCT (mmol/L)	SCT at time of diagnosis (mmol/L)	CFTR Gene 1	CFTR Gene 2	Age at CF Diagnosis (years)	Reason for Diagnosis
1	92.3	54	65	F508DEL	S1455x	1.1	SCT (65 mmol/L)
2	111.8	55	68	R553X	R117H 5T/7T	4.8	SCT (68 mmol/L)
3*	122.1	53	61	F508DEL	W1282C	8.2	SCT (61 mmol/L)^∋^
4	162	43	42	F508DEL	F191V	8.4	Genetics Re-classified
5	61.3	32	55	F508DEL	R334Q	7.5	Clinical Symptoms
6	43.5	41	49	F508DEL	R117H 7T/9T	6.6	Clinical Symptoms
7*	143.7	37	57	F508DEL	W1282C	2.8	Clinical Symptoms
8	67.3	47	66	3120 + 1G > A	I618T	2.9	SCT (66 mmol/L)
9	75.6	37	60	F508DEL	2789 + 2insA	0.4	SCT (60 mmol/L)
10	98.6	39	39	F508DEL	F191V	0.18	Expanded Genetics
11	Unsat*^Δ^*	37	41	F508DEL	V456A	0.28	Expanded Genetics

* Patients 3 and 7 are siblings.

^∋^ Patient 3 had stool elastase <200 µg/g.

^Δ^ Abbreviated for “unsaturated.” In Alabama, infants with insufficient blood detected on the NBS panel will automatically receive the limited DNA analysis panel to limit missing potential diagnosis of CF in the infant.

### Biochemical biomarkers of cf diagnosis in CRMS sub-groups

**Figure 2:**
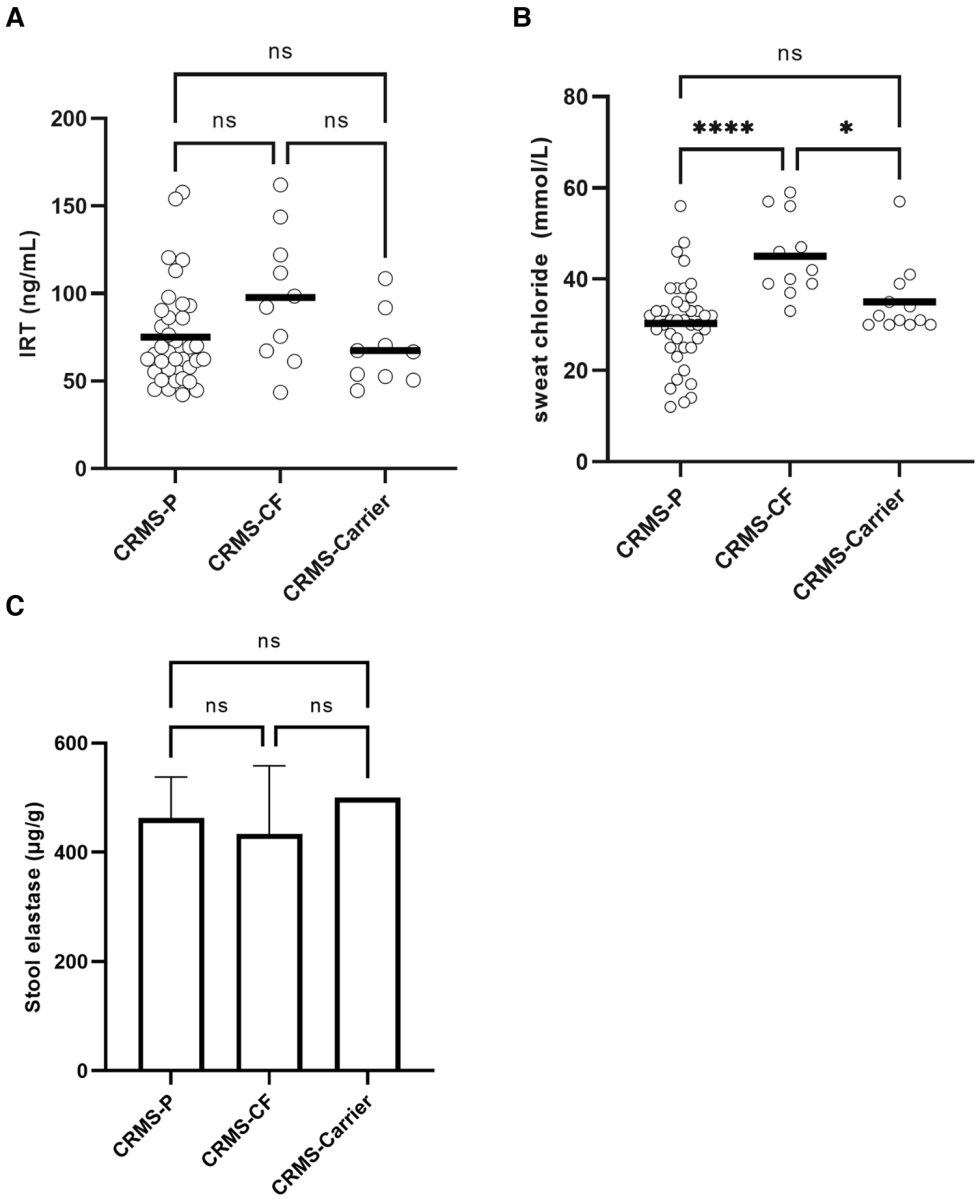
Biochemical biomarkers in CRMS sub-groups for IRT, initial SCT, and stool elastase. (**A**) Initial IRT results from NBS. A minority of patients had a result higher than the upper limit of quantitation, or an initial result that could not be determined. Comparisons are made between CRMS subgroups. (**B**) First successful SCT results by subgroup. First quantifiable results of SCT are shown. ****p <* *0.0001, *p =* *0.028. (**C**) Stool elastase results. The upper limit of quantitation of 500 µg/g stool is represented on the graph as 500 µg/g and was the result in 66% (25/38) available samples (including 6/10 CRMS-CF samples, 5/5 CRMS-Carrier samples, and 14/23 CRMS-*P* samples).

In contrast to infants who were diagnosed with CF after completing the initial NBS process, infants initially diagnosed as CRMS and then transitioned later to a diagnosis of CF (CRMS-CF) had a higher, non-significant difference in the IRT value (97.8 ng/ml) on NBS compared to CRMS-*P* (75.2 ng/mL, *p *= 0.089) or CRMS-Carrier (67.4 ng/ml, *p *= 0.072) groups (Figure 2). The CRMS-CF group had a statistically significant elevation of their first SCT value (45 mmol/L) compared to CRMS-*P* (30 mmol/L, *p *<* *0.0001) or CRMS-Carrier (35, *p *=* *0.0279) groups. Because IRT was elevated in the CRMS-CF group (albeit non-significantly elevated), as was first SCT, we assessed inclusion of both IRT and SCT in the model to assess prediction of CF transition. First successful SCT was found to predict CRMS-*P* vs. CRMS-CF (*p *=* *0.001) and CRMS-CF vs. CRMS-Carrier (*p *=* *0.0149). Including IRT value does not further increase ability to predict CF transition. When we evaluated the trajectory of SCT repeated measures over time between the CRMS-CF and CRMS-*P* groups (the CRMS-Carrier having insufficient repeated measures to evaluate), we found that SCT concentrations increase with age (about 0.79 for every additional year of age, *p *=* *0.014) over both groups. The mean first SCT concentration in the CRMS-CF group is higher than for CRMS-*P* and the CRMS-CF group increases more with age (1.23 mmol/L/year) compared to those in the CRMS-*P* group (0.49 mmol/L/year, *p *=* *0.206). Stool elastase was collected from 38 children in the CRMS subgroups. Only one child had elastase <200 µg/g (transitioned to CRMS-CF) and otherwise no differences were appreciated between any of the subgroups.

### Microbiology of CRMS subgroups

**Figure 3:**
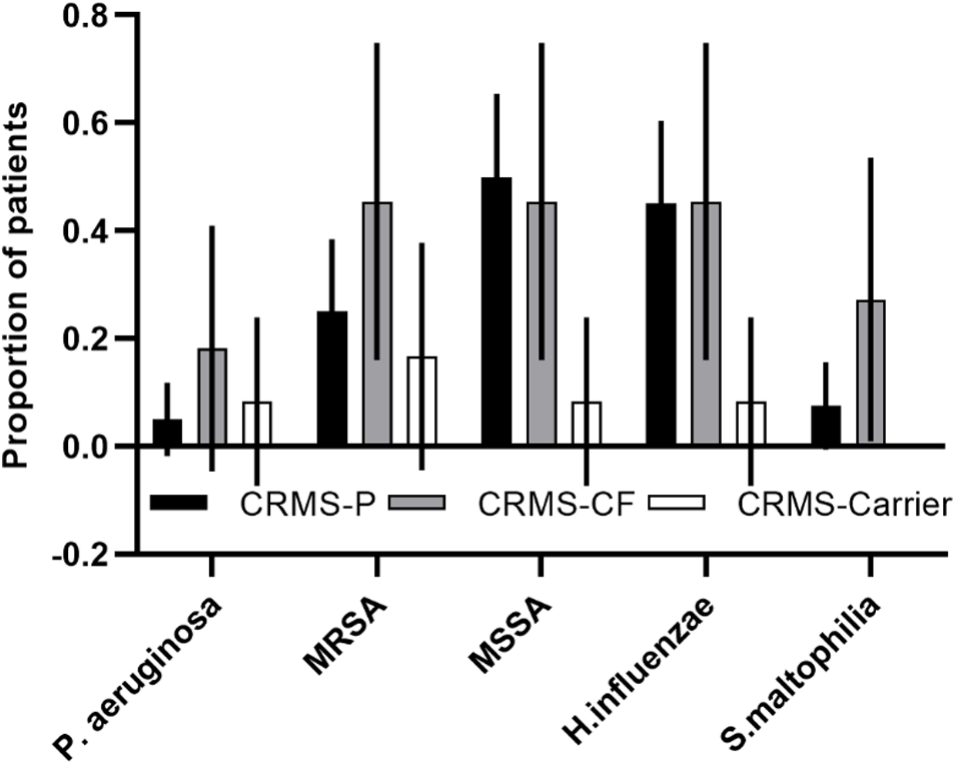
CF-related pathogens isolated in the CRMS subgroups. Proportion of children in each group that ever isolated the described bacteria are shown for each subgroup. 95% confidence intervals are shown for each.

A majority (76%) of children in the CRMS subgroups had a result for at least one sputum swab culture. Culture results were collected from all children with an initial designation of CRMS, with any cultures obtained after transition to a diagnosis of CF excluded. We had at least one culture in all of the children in the CRMS-CF group (range 1–16), 80% of children in the CRMS-*P* group (range 1–22), and 42% of children in the CRMS-Carrier group (range 1–2). There was a non-significant difference in the total number of cultures per patient collected between the CRMS-CF (mean* *=* *5.5) vs. CRMS-*P* (mean* *=* *3.4) groups. Results showing oropharyngeal flora and probable environmental contaminants were excluded, and number of unique pathogenic species ever cultured were normalized to number of cultures per child. Those in the CRMS-CF group had a slightly higher, non-significant number of unique species (0.75 species/culture) identified on microbiology compared to CRMS-Carrier (0.41 species/culture) and CRMS-*P* (0.61 species/culture) groups. Certain known CF-related pathogens (*Pseudomonas aeruginosa,* Methicillin-resistant *Staphylococcus aureus,* and *Stenotrophomonas maltophilia)* were isolated at least once in a greater proportion of children in the CRMS-CF group (18%, 45%, and 27%, respectively) compared to CRMS-*P* (5%, 25%, 7.5%) or CRMS-Carrier (8%, 17%, 0%) groups (Figure 3).

### Anthropomorphic measures

We examined weight-for-height, weight-for-age, and height-for-age using percentiles created by the CDC 2000 growth charts for children age 0 to 20 years old (26). These percentiles adjust for developmental differences among children in their anthropomorphic measurements by age and sex. Examining differences in these measures at first visit, we found no significant differences between CRMS-*P*, CRMS-CF, and CRMS-Carrier on weight-for-age-percentile or height-for-age-percentile, and no trend for CRMS-CF to be smaller than the other groups for these measures. When examining change over time in the anthropometric data between CRMS-CF and CRMS-*P* (there was insufficient data to evaluate CRMS-Carrier group), we found that children in the CRMS-*P* group have higher weight-for-height percentiles at first observation (59.7 [51.3,68.1] vs. 49.0 [24.0,74.0]) compared to children in the CRMS-CF group. Children in the CRMS-CF group appear to have a faster rate in growth than children in the CRMS-*P* group, but remain below the weight-for-height of those in CRMS-*P* by the time of the last observation (60.4 [43.5,77.3] vs. 67.0 [56.7,77.4]). Among children in the CRMS-CF group, each additional month is associated with an increase in weight-for-height percentile of 0.21 (*p *=* *0.01), while children in the CRMS-*P* group have a non-significant change in weight-for-height percentile (−0.33, *p *=* *0.695; difference between the two changes is 0.24, *p*=0.038). We do not see differences in their weight-for-age percentile or height-for-age percentile trajectories.

## Discussion

Our single-center analysis identified that 18% (11 of the 63 total CRMS) of all children who met CRMS criteria on initial encounter transitioned to a conclusive diagnosis with CF, with 4 of these 11 children (36%) diagnosed after the age of 6 years of age. One-third of the children who transitioned were in the low intermediate range (30–39 mmol/L) on initial SCT, whereas the trajectory of SCT progression per year on repeated measurements was significantly greater in those who transitioned than those who did not. No child who transitioned to a diagnosis of CF had an initial SCT value in the normal range. No child who transitioned to CF demonstrated initial symptoms, and most were diagnosed while still asymptomatic on the basis of sweat test in the diagnostic range on repeat testing or genetics. While there were some modest differences in growth, there were no clear thresholds for predicting transition in diagnosis on the basis of growth parameters.

Biomarkers, such as IRT or SCT, may be helpful in predicting those who will transition to a CF diagnosis. Several studies have looked into the role initial and repeat SCT values may serve in predicting which infants with CRMS may potentially transition to a CF diagnosis (19, 20, 23). In our analysis, none of those with CRMS status with initial SCT values <30 mmol/L have so far transitioned to a conclusive CF diagnosis. Of the 11 children who transitioned from CRMS to a diagnosis of CF, all of them had an initial SCT value within the intermediate range. We found that 12% (4 out of 34 total) of children with CRMS status with initial SCT values between 30 and 39 mmol/L transitioned to CF. This is in contrast to 50% (4 out of 8) with initial SCT values between 40 and 49 mmol/L and 60% (3 out of 5 total) with initial SCT in the 50–59 mmol/L who ultimately received a diagnosis of CF.

While IRT is useful for screening for CF, its utility alone to predict a transition to CF among those who initially meet CRMS criteria is low (27, 28). Despite this, our data shows that those who transitioned to a CF diagnosis had a higher IRT than the other subgroups, as well as a higher initial SCT. Furthermore, progressive increases in SCT over time may also be indicative of an eventual CF diagnosis, as has been reported previously (21, 24). Systematic evaluation of these biomarkers in multi-center studies is needed to define the risk and provide evidence for appropriate counseling and earlier diagnosis. With greater sample size, a predictive model using both IRT and initial SCT may prove useful for earlier diagnosis and should be evaluated.

Children with CRMS designation who transition to a CF diagnosis are not easily distinguishable from those who do not on the basis of clinical symptoms. Nine out of 11 of our CRMS-CF population remained pancreatic sufficient according to elastase results (and one unknown as the patient was followed elsewhere for care after diagnosis). There appears to be a modest difference in the isolation of pathogens, including CF-related pathogens before the CF diagnosis was confirmed in the CRMS-CF group. However, for most of these children, there were no concerns regarding chronic symptoms suggestive of CF prior to diagnosis. With a modestly higher isolation of CF-related pathogens identified in those who ultimately transitioned to a CF diagnosis, we speculate that appropriately powered, systematic studies of the colonization status in this population may provide greater insight into those who may need longer follow-up or more aggressive evaluation. There are also some modest differences in growth parameters. None of these present clear-cut thresholds to help with prognostic counseling.

There has been progress over the past ten years with respect to the evaluation, designation and early management of infants with CRMS (5, 23, 29). There is less clear evidence on long term management. The data from one study demonstrated that a significant proportion of asymptomatic children with CRMS reach 6 years of age in good health with normal growth, lung function and imaging and normal sweat chloride values (<30 mmol/L) and therefore are discharged from annual care at a CF Center (23). However, over one-third of children in our population transitioned to a CF diagnosis after the age of 6 years. Of these four children, two transitioned due to development of clinical symptoms and one due to repeat SCT in the diagnostic range. For populations similar to those in our study, continuing to monitor after age 6 years is warranted.

Despite the published and updated guidance, there is variation in practice and management for children with CRMS designation, possibly due to the lack of clear evidence supporting risk assessment and counseling. One of the unfortunate consequences of this inconsistent management is the potential for patients to be lost to follow-up. At our center, we identified 12 CRMS children that had been lost to follow-up between the years 2008 to 2019. In 2019, our institution implemented a center wide protocol based on updated guidelines, with our center re-engaging nearly 50% of that patient population for continued care. Given the data found in our analysis, we would emphasize the importance of consistency amongst CF providers at a center in ensuring this patient population is managed appropriately. Additionally, increased anxiety is exhibited by families for conditions associated with an inconclusive diagnosis, which can be ameliorated by providing clear information to families (30). While there is no internationally accepted consensus on the optimal approach to providing families with appropriate counseling for CRMS, there is evidence to support that CF Centers should incorporate counseling sessions with annual follow-ups to provide the family with awareness regarding any potential risk of transition to CF.

There are several limitations to our study. This study was conducted using retrospective chart review over a more than a decade, during which our institution underwent several changes in medical record keeping. Our analysis was conducted for a single state with one CF Center and therefore is associated with a small sample size. Over the 12 years of study evaluation, there were many different providers providing care, as well as changes in evidence-based guidelines for management. In our study, we retrospectively categorized each participant according to the updated 2017 diagnostic criteria to improve consistency across all years of evaluation. For many outcomes of interest, the practice variation resulted in missing data. For example, we were unable to identify one or more repeat SCT results for 8 (12.7%) of the patients, and other data such as culture results, fecal elastase, and growth parameters. Additionally, other states may use a different CF NBS algorithm or a different mutation screening panel, which may yield different results.

In conclusion, we describe a long-term, retrospective study of children designated CRMS and followed thereafter at an accredited CF Center. Our data complements previous studies that have noted a transition from CRMS designation to a conclusive CF diagnosis, while providing some important caveats to prior publications. Our results suggest that all infants with IRT+/DNA+ and a SCT within the intermediate range between 30 and 59 mmol/L need to be monitored closely for CF with strong consideration for repeat SCT, expanded genotyping, and microbiologic surveillance. Furthermore, children with elevated initial SCT, as well as those whose SCT is increasing in childhood, are likely higher risk for transition to a CF diagnosis. A multi-center effort to systematically study this population over childhood with harmonization of diagnostic criteria, evaluation, and follow-up should be initiated. This study should include assessment of current and novel biomarkers to better predict CFTR dysfunction as early as possible, especially with the advent of highly effective modulator therapy which may delay or prevent manifestations of CF disease.

## Data Availability

The original contributions presented in the study are included in the article/**Supplementary materials**, further inquiries can be directed to the corresponding author.

## Author disclaimer

This statement is to certify that all Authors have seen and approved the manuscript being submitted. We warrant that the article is the Authors’ original work. We warrant that the article has not received prior publication and is not under consideration for publication elsewhere. On behalf of all Co-Authors, the corresponding Author shall bear full responsibility for the submission. This research has not been submitted for publication nor has it been published in whole or in part elsewhere. We attest to the fact that all Authors listed on the title page have contributed significantly to the work, have read the manuscript, attest to the validity and legitimacy of the data and its interpretation. The work for this study was performed at the University of Alabama at Birmingham. None of the Authors have any conflicts of interest to disclose. No financial support was provided for development of this study.
