# Supplementary material for: Outcomes of children with cystic fibrosis screen positive, inconclusive diagnosis/CFTR related metabolic syndrome
Source: Front Pediatr. 2023 Mar 9;11:1127659. doi: 10.3389/fped.2023.1127659 (PMC10034052; doi:10.3389/fped.2023.1127659)
Supplement: Supplementary file 1 [file Table1.docx]

|  | **Age of 1^st^ SCT (y)** | **Mean 1^st^ SCT (mmol/L)** | **Age of Last SCT**  **(y)** | **Mean Last SCT**  **(mmol/L)** | **Genotype 1** | **Genotype 2** |
| --- | --- | --- | --- | --- | --- | --- |
| **Initial SCT <30 mmol/L** | | | | | | |
| **CRMS-P** | 0.09 | 12 | 0.09 | 12 | F508del | D1270N |
| **CRMS-P** | 0.09 | 25 | 11.9 | 42 | F508del | R117H 7T/9T |
| **CRMS-P** | 0.17 | 27 | 0.5 | 34 | R117H 7T/9T | F508del |
| **CRMS-P** | 0.1 | 20 | 0.98 | 20 | F508del | R117H 7T/9T |
| **CRMS-P** | 0.04 | 29 | 0.04 | 29 | F508del | R117H 7T/9T |
| **CRMS-P** | 0.11 | 14 | 0.62 | 17 | F508del | D1152H |
| **CRMS-P** | 0.09 | 16 | 7.75 | 35 | F508del | 5T;TG11/9T;TG10 |
| **CRMS-P** | 0.29 | 28 | 6.13 | 17 | R117H 7T/9T | F508del |
| **CRMS-P** | 0.08 | 29 | 1.15 | 30 | F508del | R117H 7T |
| **CRMS-P** | 0.06 | 23 | 0.48 | 13 | F508del | R117H 7T/9T |
| **CRMS-P** | 0.19 | 25 | 3.38 | 27 | F508del | R117H 7T/9T |
| **CRMS-P** | 0.04 | 18 | 0.04 | 18 | F508del | R117H 7T/9T |
| **CRMS-P** | 0.14 | 13 | 0.14 | 13 | F508del | 5T;TG11 |
| **CRMS-P** | 0.2 | 17 | 0.2 | 17 | F508del | 5T;TG11 |
| **CRMS-P** | 0.03 | 27 | 0.17 | 22 | F508del | D1152H |
| **CRMS-P** | 0.05 | 25 | 0.32 | 33 | F508del | R117H 7T/9T |
| **Initial SCT 30-59 mmol/L** | | | | | | |
| **CRMS-P** | 0.09 | 56 | 2.24 | 37 | F508del | Y1032C |
| **CRMS-P** | 0.04 | 31 | 0.04 | 31 | S549N | D1270N |
| **CRMS-P** | 0.11 | 31 | 0.24 | 27 | R117H | F508del |
| **CRMS-P** | 0.1 | 31 | 0.18 | 25 | F508del | 5T;TG11/9T;TG10 |
| **CRMS-P** | 0.1 | 33 | 1.82 | 26 | F508del | 5T;TG12/9T;TG10 |
| **CRMS-P** | 0.08 | 30 | 0.08 | 30 | G542X* |  |
| **CRMS-P** | 0.08 | 30 | 10.51 | 34 | F508del | Y1014C |
| **CRMS-P** | 0.08 | 33 | 0.56 | 31 | F508del | 5T;TG12/9T;TG10 |
| **CRMS-P** | 0.08 | 38 | 0.6 | 22 | F508del | M265R |
| **CRMS-P** | 0.12 | 32 | 5.12 | 17 | R117H 5T/7T | 5T;TG11/7T;TG10 |
| **CRMS-P** | 0.05 | 38 | 6.75 | 26 | 621+1G->T | 5T;TG13 |
| **CRMS-P** | 0.12 | 48 | 8.47 | 49 | 3120+1G>A | F1099L |
| **CRMS-P** | 0.05 | 46 | 8.45 | 38 | F508del | R117H 7T/9T |
| **CRMS-P** | 0.07 | 33 | 7.76 | 29 | F508del | 5T;TG11 |
| **CRMS-P** | 0.16 | 38 | 2.38 | 42 | F508del | R117H 7T/9T |
| **CRMS-P** | 0.08 | 34 | 2.98 | 39 | F508del | p.l1328T, p.A1374G |
| **CRMS-P** | 0.16 | 44 | 2.09 | 35 | F508del | 5T;TG12 |
| **CRMS-P** | 0.06 | 35 | 0.19 | 26 | F508del | 5T;TG13 |
| **CRMS-P** | 0.06 | 36 | 0.06 | 36 | F508del | 5T;TG11 |
| **CRMS-P** | 0.06 | 32 | 1.66 | 43 | F508del* |  |
| **CRMS-P** | 0.06 | 32 | 0.89 | 46 | F508del | R117H 7T/9T |
| **CRMS-P** | 0.16 | 30 | 1.06 | 34 | R1162X | 5T;TG12 |
| **CRMS-P** | 0.12 | 39 | 0.22 | 40 | F508del | 5T;TG11 |
| **CRMS-P** | 0.07 | 33 | 0.14 | 33 | F508del* |  |
| **CRMS-Carrier** | 0.07 | 30 | 0.17 | 23 | G551D |  |
| **CRMS-Carrier** | 0.01 | 39 | 0.48 | 17 | F508del |  |
| **CRMS-Carrier** | 0.09 | 35 | 0.26 | 19 | F508del |  |
| **CRMS-Carrier** | 0.3 | 31 | 2.44 | 23 | R117H 7T/7T |  |
| **CRMS-Carrier** | 0.08 | 30 | 0.39 | 18 | F508del |  |
| **CRMS-Carrier** | 0.16 | 57 | 0.2 | 11 | 5T;TG13 |  |
| **CRMS-Carrier** | 0.04 | 30 | 2.36 | 19 | 3120+1G>A |  |
| **CRMS-Carrier** | 0.15 | 30 | 0.5 | 27 | F508del |  |
| **CRMS-Carrier** | 0.12 | 34 | 0.23 | 13 | F508del |  |
| **CRMS-Carrier** | 0.04 | 41 | 0.1 | 24 | 1717-1G>A |  |
| **CRMS-Carrier** | 0.06 | 32 | 0.23 | 24 | F508del |  |
| **CRMS-Carrier** | 0.08 | 31 | 0.33 | 16 | R117H 7T/7T |  |
| **CRMS-CF** | 0.1 | 56 | 1.12 | 65 | F508del | S1455X |
| **CRMS-CF** | 0.09 | 59 | 11.83 | 39 | R553X | R117H 5T/7T |
| **CRMS-CF** | 0.08 | 57 | 8.2 | 61 | F508del | W1282C |
| **CRMS-CF** | 0.07 | 46 | 3.21 | 25 | F508del | F191V |
| **CRMS-CF** | 0.08 | 33 | 7.45 | 55 | F508del | R334Q |
| **CRMS-CF** | 0.07 | 42 | 6.6 | 49 | F508del | R117H 7T/9T |
| **CRMS-CF** | 0.1 | 40 | 2.75 | 57 | F508del | W1282C |
| **CRMS-CF** | 0.1 | 47 | 3.86 | 66 | 3120+1G>A | I618T |
| **CRMS-CF** | 0.08 | 39 | 0.41 | 60 | F508del | 2789+2insA |
| **CRMS-CF** | 0.22 | 37 | 0.52 | 41 | F508del | V456A |
| **CRMS-CF** | 0.05 | 39 | 0.05 | 39 | F508del | F191V |

*CRMS-P on the basis of persistent intermediate SCT.
